# Connexin30 and Connexin43 show a time-of-day dependent expression in the mouse suprachiasmatic nucleus and modulate rhythmic locomotor activity in the context of chronodisruption

**DOI:** 10.1186/s12964-019-0370-2

**Published:** 2019-06-11

**Authors:** Amira A. H. Ali, Anna Stahr, Marc Ingenwerth, Martin Theis, Christian Steinhäuser, Charlotte von Gall

**Affiliations:** 1 Institute of Anatomy II, Medical Faculty, Heinrich-Heine-University, Merowinger Platz 1A, 40225 Düsseldorf, Germany; 2 Institute of Pathology, Medical Faculty, University of Duisburg–Essen, Hufelandstrasse 55, 45147 Essen, Germany; 3 Institute of Cellular Neurosciences, Medical Faculty, University Bonn, Sigmund Freud Str. 25, 53105 Bonn, Germany

**Keywords:** Circadian rhythm, SCN, Jet lag, Constant darkness, Constant light, cFOS, Entrainment

## Abstract

**Background:**

The astroglial connexins Cx30 and Cx43 contribute to many important CNS functions including cognitive behaviour, motoric capacity and regulation of the sleep-wake cycle. The sleep wake cycle, is controlled by the circadian system. The central circadian rhythm generator resides in the suprachiasmatic nucleus (SCN). SCN neurons are tightly coupled in order to generate a coherent circadian rhythm. The SCN receives excitatory glutamatergic input from the retina which mediates entrainment of the circadian system to the environmental light-dark cycle. Connexins play an important role in electric coupling of SCN neurons and astrocytic-neuronal signalling that regulates rhythmic SCN neuronal activity. However, little is known about the regulation of Cx30 and Cx43 expression in the SCN, and the role of these connexins in light entrainment of the circadian system and in circadian rhythm generation.

**Methods:**

We analysed time-of-day dependent as well as circadian expression of Cx30 and Cx43 mRNA and protein in the mouse SCN by means of qPCR and immunohistochemistry. Moreover, we analysed rhythmic spontaneous locomotor activity in mice with a targeted deletion of Cx30 and astrocyte specific deletion of Cx43 (DKO) in different light regimes by means of on-cage infrared detectors.

**Results:**

Fluctuation of Cx30 protein expression is strongly dependent on the light-dark cycle whereas fluctuation of Cx43 protein expression persisted in constant darkness. DKO mice entrained to the light-dark cycle. However, re-entrainment after a phase delay was slightly impaired in DKO mice. Surprisingly, DKO mice were more resilient to chronodisruption.

**Conclusion:**

Circadian fluctuation of Cx30 and Cx43 protein expression in the SCN is differently regulated. Cx30 and astroglial Cx43 play a role in rhythm stability and re-entrainment under challenging conditions.

**Electronic supplementary material:**

The online version of this article (10.1186/s12964-019-0370-2) contains supplementary material, which is available to authorized users.

## Introduction

Gap junctions represent direct cell-to-cell communications, which allow trafficking of a wide variety of small molecules (< 1.5 kDa) including ions, neurotransmitters, and metabolites such as glucose between the cytoplasm of adjacent cells, and hence regulating intercellular metabolic and electric coupling. A gap junction is formed of Connexin (Cx) proteins arranged as two opposed leakless hexameric hemichannels (connexons) [1–3]. Twenty different Cx proteins are expressed in mice and named with respect to their molecular weight (in kDa) [4], including Cx23, Cx26, Cx29, Cx30, Cx30.2, Cx31, Cx32, Cx33, Cx37, Cx36, Cx39, Cx40, Cx43, Cx45, Cx46, Cx47, Cx50 and Cx57 [5]. Among all cell types in the CNS, astrocytes show the highest Cx expression, in particular Cx43 and Cx30. Although these Cx proteins are widely expressed in the brain, their expression shows region-dependent variation, e.g. in the hypothalamus, thalamus, and hippocampus [6–8], which may suggest special roles of Cx isoforms in the function of these prosencephalic structures. Recent studies showed that astroglial Cx43 and/or Cx30 contribute to important CNS functions such as neuroprotection [9], neurogenesis [10–12], synaptic strength [13–16], K^+^ spatial buffering [16], integrity of blood brain barrier [17], cognitive behaviour [18], fear memory consolidation [19], explorative behaviour, motoric capacity and brain neurochemistry [20] and glucose sensing [21]. Recently, Cx43 has been shown to contribute to the regulation of the sleep-wake cycles by astrocyte-neuron metabolic interactions at the level of the lateral hypothalamus [22]. However, in mice lacking astrocytic Cx43, a 50% residual interastrocytic coupling has been observed, while Cx30 expression was significantly upregulated, suggesting a compensatory mechanism [15]. Thus, for a complete disruption of astrocytic coupling, Cx30- and astroglial-specific Cx43 double knockout mice (DKO) mice are a well-established model [10, 16, 17, 23]. The suprachiasmatic nucleus (SCN) of the hypothalamus is the master pacemaker of the circadian system that orchestrates circadian rhythms in behaviour, metabolic and physiological processes as well as endocrine and neuronal function [24]. Under natural and laboratory conditions, rhythmic body functions are entrained to the environmental light-dark cycle. However, rhythms, which are controlled by the molecular clockwork, consisting of transcriptional/translational feedback loops of clock genes [25], persist even in the absence of environmental cues such as in constant darkness. Light is an important time cue (Zeitgeber) to entrain the molecular clockwork [26]. Excitatory glutamatergic inputs from retinal ganglion cells mediate entrainment of the circadian system to the environmental light-dark cycle (Kim and Dudek, 1991; Castel et al., 1993; Mintz et al., 1999). However, exposure to constant light results in a disruption of circadian rhythms. Within the SCN, neuronal gap junctions are important for electric coupling, which is crucial to generate a coherent circadian rhythm of neuronal firing [27]. Importantly, deletion of neural connexin Cx36 significantly impairs the SCN electric coupling resulting in changes of circadian period length [28, 29]. Moreover, astrocytic-neuronal signalling regulates rhythmic SCN neuronal activity and thus circadian rhythm generation [30]. However, very little is known about the expression and the role of the astrocytic connexins Cx30 and Cx43 in the SCN. Therefore, in this study, we analysed the expression of *Cx30* and *Cx43* mRNA by means of real time PCR as well as Cx30 and Cx43 proteins by means of immunohistochemistry in mouse SCN at different time points during the 24 h cycle under different light regimes. Moreover, to determine the role of interastrocytic coupling for entrainment and circadian rhythm generation and stability, we analysed spontaneous locomotor activity in a jet lag paradigm, in constant darkness (DD) and in a paradigm for chronodisruption (constant light) in Cx30- and astroglial-specific Cx43 double knockout mice (DKO). Expression of *Cx30* and *Cx43* mRNA in the SCN exhibits a time-of-day-dependent variation with a peak during the early dark phase. However, this fluctuation was absent when the mice were kept in constant darkness, suggesting that rhythmic expression of *Cx30* and *Cx43* mRNA is driven by the light/dark cycle. Cx30- and Cx43-immunoreaction (Ir) in the SCN exhibit a time-of-day-dependent variation with a peak during the light phase. In constant darkness, Cx43- but not Cx30-Ir variation persisted. Moreover, in constant light, Cx30 was constitutively high whereas Cx43-Ir was constitutively low. This suggests a different regulation of Cx30 and Cx43 in the SCN. In DKO mice, spontaneous locomotor activity was entrained to the light/dark cycle. However, DKO mice re-entrained not as fast as wildtype (WT) littermates in response to a phase delay in an experimental jet lag paradigm. Furthermore, after constant light exposure, DKO mice showed a significantly higher activity and circadian rhythm amplitudes as compared to WT mice. In summary, the expression of Cx30 and Cx43 in the SCN is predominantly regulated by the light/dark cycle and deletion of Cx30 in combination with astroglial deletion of Cx43 affects re-entrainment of circadian rhythms and modulates resilience to chronodisruption.

## Material and methods

### Animals

Breeding and experiments were performed at the animal facility of the Medical Faculty, Heinrich-Heine University, Düsseldorf, Germany. During the experiments, mice were housed in single cages in light- and sound-proof cabinets with automatic time switch (Beast master, Germany). The light intensity during the light phase was 400 lx. All mice had free access to food and water.

Male C57Bl/6 mice (12–15 weeks old) were kept under different light conditions. To analyse the influence of a normal light-dark-cycle on the expression of Cx30 and Cx43, mice were kept under 12 h light and 12 h darkness (12:12 LD) (light on at 6:00 am = Zeitgeber time (ZT) 00), animals were sacrificed every 4 h at ZT02, ZT06, ZT10, ZT14, ZT18 and ZT22. To analyse the role of internal clock on the expression of Cx30 and Cx43, animals were housed under constant darkness (DD) for at least 38 h and were sacrificed at the circadian time points (CT, CT00 = lights on in the former light phase, 6 am) CT02, CT06, CT10, CT14, CT18 and CT22. To evaluate the impact of constant light on the expression of Cx30 and Cx43, animals were housed for 14 days under constant light conditions and sacrificed at the Disrupted Time points (DT, DT00 = 6:00 am) DT02, DT06, DT10, DT14, DT18 and DT22. *n* = 3 mice at each time point.

DKO male mice (8–12 weeks old) with conditional deletion of Cx43 in astrocytes and conventional deletion of Cx30 (Cx43^fl/fl^:hGFAP-Cre/Cx30^−/−^) and WT (Cx43^+/+^/Cx30^+/+^) were used for analysis of spontaneous locomotor activity [16]. Genotype was confirmed by PCR.

### Analysis of spontaneous locomotor activity

Spontaneous locomotor activity was continuously recorded by using on-cage infrared movement detectors linked to a monitoring system (Mouse-E-Motion, Germany). Actograms, activity onset, activity profiles, chi-square periodograms and amplitude of circadian rhythmicity were analysed by Clocklab software (Actimetrics, Wilmette, USA). Male DKO and WT mice (*n* = 6 mice of each genotype) were housed in individual standard cages with free access to food and water. For experimental jet lag, mice were kept for 3 weeks in 12:12 LD (lights on at 6 am, 12.1), then the light cycle was advanced by 6 h (− 6, lights on at 00:00). After 3 weeks under these conditions, the light cycle was again delayed by 6 h (+ 6, lights on at 6:00 am) and kept for 3 weeks under these conditions. After confirming complete re-entrainment, mice were kept for additional 3 weeks in constant darkness (DD), followed by 3 weeks in constant light (LL) and another 3 weeks in 12:12 LD (lights on at 6 am, 12.2). Phase angle of entrainment is defined as onset of activity relative to lights off. Onset of activity after lights off is expressed as a positive phase angle of entrainment, onset of activity prior to lights off is expressed as a negative phase angle. A delayed positive phase angle reflects a delayed onset of activity, a negative phase angle an advanced onset of activity.

### Analysis of mRNA-expression

Adult C57Bl/6 mice (*n* = 3 each time point) were killed by isoflurane. Coronal sections were prepared by using a stainless steel adult mouse brain matrix for coronal sections (Zivic Instruments). In slices between bregma − 0.08 to − 1.5 mm the SCN region (according to Allen Mouse Brain Atlas (2004)) was dissected using a 1.0 mm inner diameter stainless steel punch needle. The dissected SCN were immediately frozen and stored at − 80 °C. Total RNA was isolated using RNeasy Lipid Tissue Mini Kit (Qiagen) according to the manufacturer’s protocol. cDNA was prepared by using QuantiTect Reverse Transcription Kit (Qiagen). Quantitative real-time PCR for Cx43 (Forward: CCCGAACTCTCCTTTTCCTT; Reverse: TGGGCACCTCTCTTTCACTT) and Cx30 (Forward: TTGCAGAGGGATTTTGCAG; Reverse: TCGTGCAGGCTTATTCTGAGT) as well as housekeeping gene (Rn18S: Forward: TTCCTTCCGGGCCTTCTCTA; Reverse: TTGGCAAATGCTTTCGCTC) were performed using KAPA SYBR FAST qPCR Kit Master Mix ABI Prism (KAPA Biosystems) in an ABI StepOne™ Plus Real-Time PCR System (Applied Biosystems) with the following PCR program: activation at 95 °C for 5 min followed by 40 cycles of denaturation at 95 °C for 3 s and amplification as well as annealing at 60 °C for 20 s. A standard curve of each primer set was used for calculation of primer efficiency. Relative Cx30 and Cx43 mRNA expression levels to RN18S in the SCN of C57Bl/6 mice at six different time points under the different light conditions was calculated using Pfaffl method [31].

### Tissue processing and immunohistochemistry

Mice were deeply anaesthetized using a ketamine:xylazine mixture (100 mg:10 mg/kg body weight, respectively) and transcardially perfused with 0.9% NaCl followed by 4% paraformaldehyde. Brains were prepared and post fixed in 4% paraformaldehyde for 24 h and cryoprotected in 20% sucrose for another 24 h. Brains were sectioned coronally (30 μm thickness) by using a cryomicrotome (Reichert-Jung) in series of 4 sections. Immunohistochemistry was perfomed with free-floating brain slices. Slices were permeabilized with washing buffer containing phosphate buffered saline (PBS) and 0.2% Triton-X 100. For quenching endogenous peroxidase activity, slices were incubated in 0.24% H_2_O_2_ for 30 min at room temperature. After washing, slices were incubated for one hour with 5% normal goat serum in PBS-T, then incubated with a primary antibody (see Table 1) overnight at 4 °C. Binding of the primary antibodies was visualized using a biotin-conjugated goat anti-rabbit IgG antibody (1:500, Vector Laboratories, BA-1000) and the avidin-biotin-peroxidase reagent (Vectastain ABC kit, Vector Laboratories) followed by an incubation in 3.3’-diaminobenzidine (Sigma-Aldrich) for 10 min. Slices were rinsed with PBS, mounted on slides, air-dried, and cover slipped with Entellan (Merck Millipore).

**Table 1:** List of primary antibodies

Antibody	Company and order-number	Dilution
Rabbit anti-Cx43	Thermo Fisher, 71–0700	1:250
Rabbit anti-Cx30	Thermo Fisher, 71–2200	1:1500
Rabbit anti-cFOS	Santa Cruz sc-522,200	1:500

The specificity of Cx30- and Cx43-antibody was confirmed in DKO mice. Cx30- and Cx43-Ir were strongly reduced in the entire brain of DKO mice, consistent with the global knockout for Cx30. The Cx43-Ir was strongly reduced in the brain (Additional file 1: Figure S1A, C) and in the SCN (Additional file 1: Figure S1B, D) of DKO mice.

### Image analysis

Microphotographs were taken under bright-field illumination using a BZ-9000 Microscope (Keyence, Japan). Image analysis was performed using Image J software (https://imagej.nih.gov/ij/) by an observer blinded to the experimental condition. Three sections of the intermediate aspect of the SCN per mouse were analysed and averaged. The microscope settings, including light intensity and exposure time, were kept constant during all image acquisitions. Cx- and cFOS-Ir were analyzed as described previously [32, 33]. The optical density of Cx Ir in the SCN region was measured above the threshold in the cell-free neuropil. Data are expressed as the percentage of area of the entire SCN covered by Cx-Ir. For analysis of cFOS-Ir, the background staining in cell-free neuropil was used to define the lower threshold. The SCN region was delineated, and all cell nuclei showing a cFOS-Ir exceeding the threshold within this region were counted.

### Statistical analysis

The statistical analysis was performed using the statistical GraphPad Prism software (GraphPad Software, Inc.). Data are presented as mean ± SEM. Differences among more than two groups (e.g. parameters at different time points) were analyzed by one-way ANOVA followed by Tukey’s posthoc test for multiple comparison. Differences between two groups (e.g. parameters in two genotypes) were analyzed by F-test for analysis of variance followed by unpaired T-test (if variances were not different) or Mann-Whitney-U Test (if variances were different). Differences between two conditions in one experimental group (e.g. before and after exposition to different light regimes) were analyzed by paired T-test. *P* value < 0.05 was considered statistically significant.

## Results

### *Cx30* and *Cx43* mRNA relative expression levels under different light conditions

**Fig. 1:**
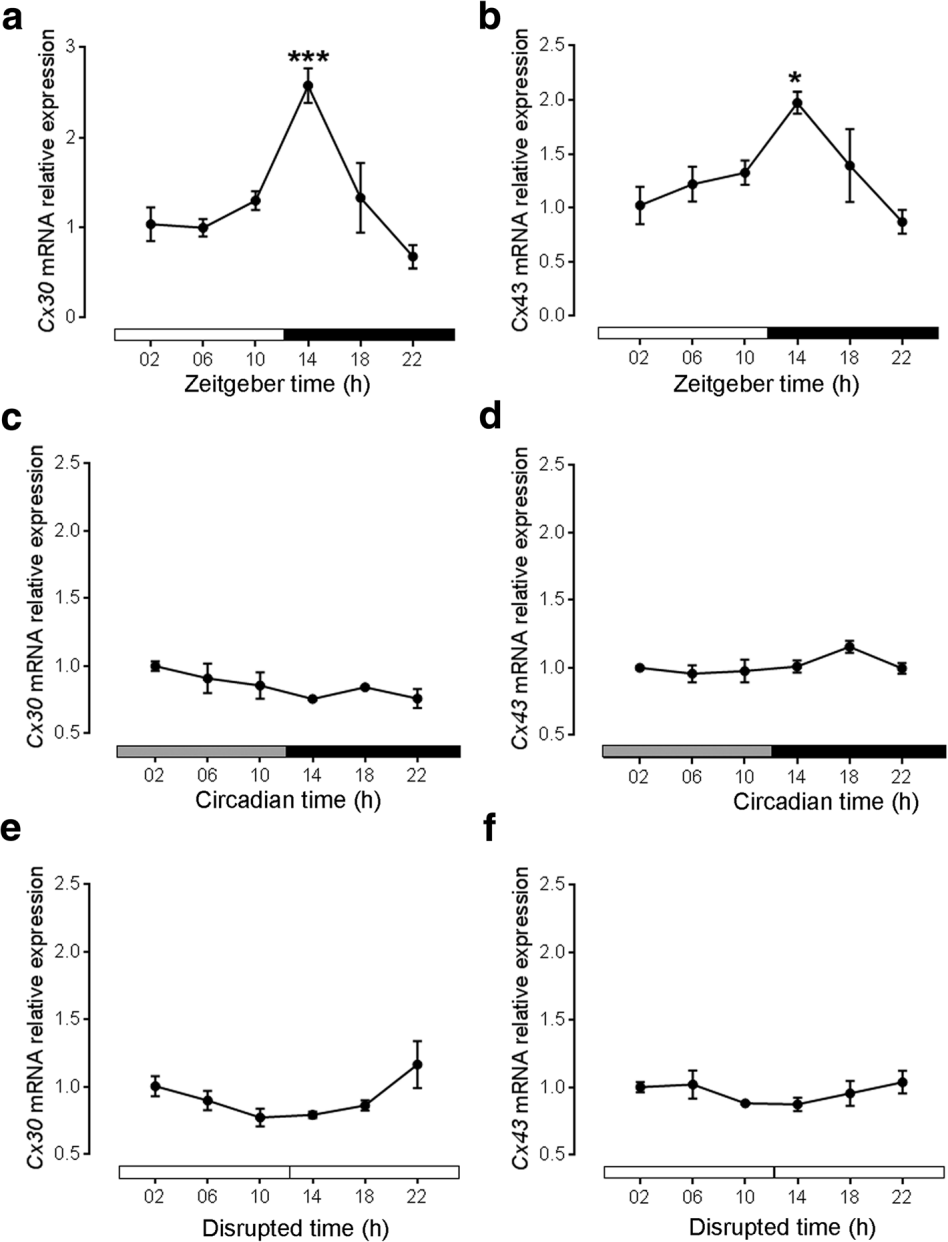
Relative expression profile of *Cx30* and *Cx43* mRNA levels under different light regimes. Relative *Cx30* (**a**) and *Cx43* (**b**) mRNA expression levels in the SCN of C57Bl/6 mice at six different time points in 12:12 LD. White bars show light phase, black bars show dark phase. Relative *Cx30* (**c**) and *Cx43* (**d**) mRNA expression levels in the SCN of C57Bl/6 mice at six different time points in constant darkness. Grey bars show former light phase, black bars show former dark phase. Relative *Cx30* (**e**) and *Cx43* (**f**) mRNA expression levels to RN18S in the SCN of C57Bl/6 mice at six different time points in constant light. Data are expressed as mean +/− SEM of *n* = 3 mice per time point. Significant differences among different time points were analysed by one way ANOVA followed by Tukey’s posthoc test. Both, C*x30* (F = 10.1; *P* = 0.0006) and *Cx43* (F = 4.24; *P* = 0.02) mRNA levels were significantly different among different time points under 12:12 LD conditions. *:*p* ≤ 0.05 vs. ZT22, ***:*p* ≤ 0.001 vs. ZT22. There were no significant differences in C*x30* or *Cx43* mRNA levels among different time points under DD or LL conditions

In the SCN of C57Bl/6 mice kept in 12:12 LD, *Cx30* (Fig. 1a) and *Cx43* (Fig. 1b) mRNA relative expression levels were significantly higher during the early dark phase (ZT14) as compared to the late dark phase (ZT22). There was no significant difference in *Cx30* (Fig. 1c, e) or *Cx43* mRNA relative expression (Fig. 1d, f) when the mice were kept in constant darkness or constant light, respectively.

### Cx30- and Cx43-immunoreaction under different light conditions

#### Cx30- and Cx43-immunoreaction in the SCN under 12:12 LD conditions

**Fig. 2:**
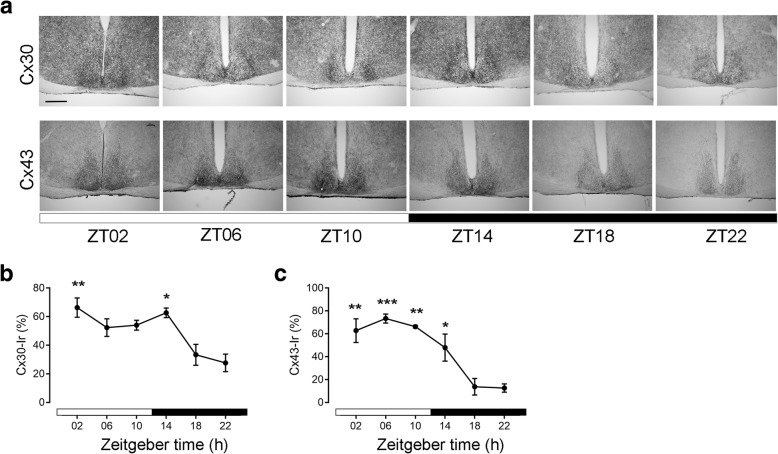
Cx43- and Cx30- immunoreaction in the SCN in 12:12 LD. **a** Representative photomicrographs of Cx30- and Cx43-Immunoreaction (Ir) in the SCN of C57Bl/6 mice kept under 12:12 LD. Scale bar: 300 μm. **b** Quantification of Cx30-Ir in the SCN. **c** Quantification of Cx43-Ir in the SCN. Data are expressed as mean +/− SEM of n = 3 mice per time point. Significant differences among different time points were analysed by one way ANOVA followed by Tukey’s posthoc test. Both, Cx30- (F = 7.41; *P* = 0.002) and Cx43- (F = 13.17; *P* = 0.0002) Ir levels were significantly different among different time points under 12:12 LD conditions. *:*P* ≤ 0.05 vs. ZT22; **:*P* ≤ 0.01; ***:*P* ≤ 0.001 vs. ZT22. White bars indicate the light phase, black bars indicate the dark phase

Time-of day dependent Cx30- and Cx43-Ir was analyzed in the SCN of C57Bl/6 mice kept in 12:12 LD. Cx30-Ir was significantly higher during the early light phase (ZT02) and the early dark phase (ZT14) than during the mid- (ZT18) or late (ZT22) dark phase (Fig. 2a, b). Cx43-Ir was significantly higher during the entire light phase and the early dark phase (ZT02-ZT14) than during the mid- (ZT18) or late (ZT22) dark phase (Fig. 2a, c). There was no obvious difference in Cx30- or Cx43- Ir between dorsomedial and ventrolateral parts of the SCN.

#### Cx30- and Cx43-immunoreaction in the SCN under constant darkness

**Fig. 3:**
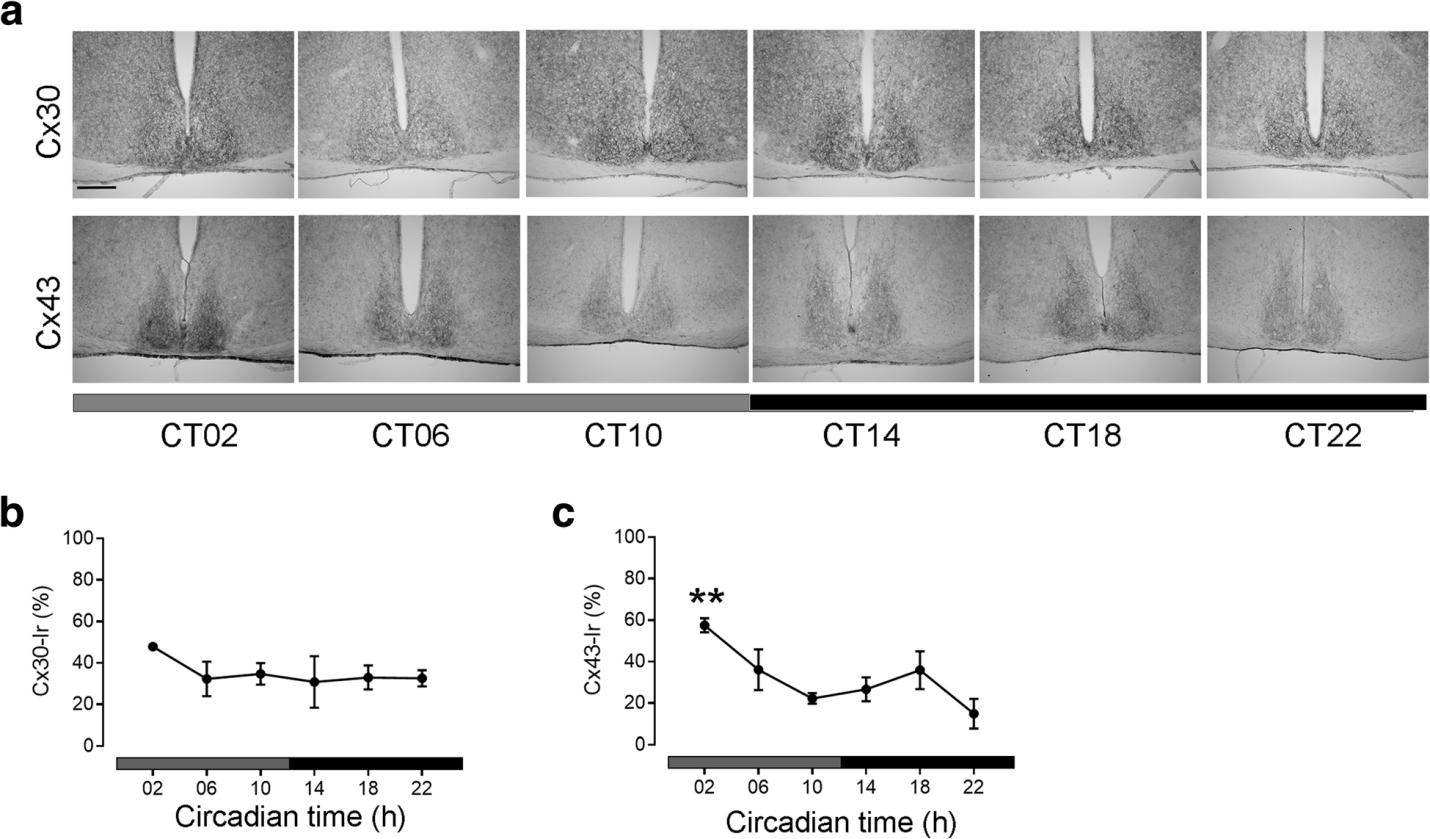
Cx43- and Cx30- immunoreaction in the SCN in constant darkness (DD). **a** Representative photomicrographs of Cx30- and Cx43-Immunoreaction (Ir) in the SCN. Scale bar: 300 μm. **b** Quantification of Cx30-Ir in SCN. **c** Quantification of Cx43-Ir in SCN. Data are expressed as mean +/− SEM of n = 3 mice per time point. Significant differences among different time points were analysed by one way ANOVA followed by Tukey’s posthoc test. Cx43- Ir levels were significantly different (F = 4.67; *P* = 0.013) among different time points under 12:12 LD conditions. **:P ≤ 0.01 vs. ZT22. Grey bars indicate the former light phase, black bars indicate the former dark phase

To analyze the light-independent circadian expression of Cx30 and Cx43 in the SCN, animals were kept for 24 h in DD. There was no significant difference in Cx30-Ir in the SCN among the different time points in DD (Fig. 3a, b). However, Cx43-Ir was significantly higher during the early subjective day (CT02) as compared to late subjective day (CT10) or the late subjective night (CT22) (Fig. 3a, c).

#### Cx30- and Cx43-immunoreaction in the SCN under constant light conditions

**Fig. 4:**
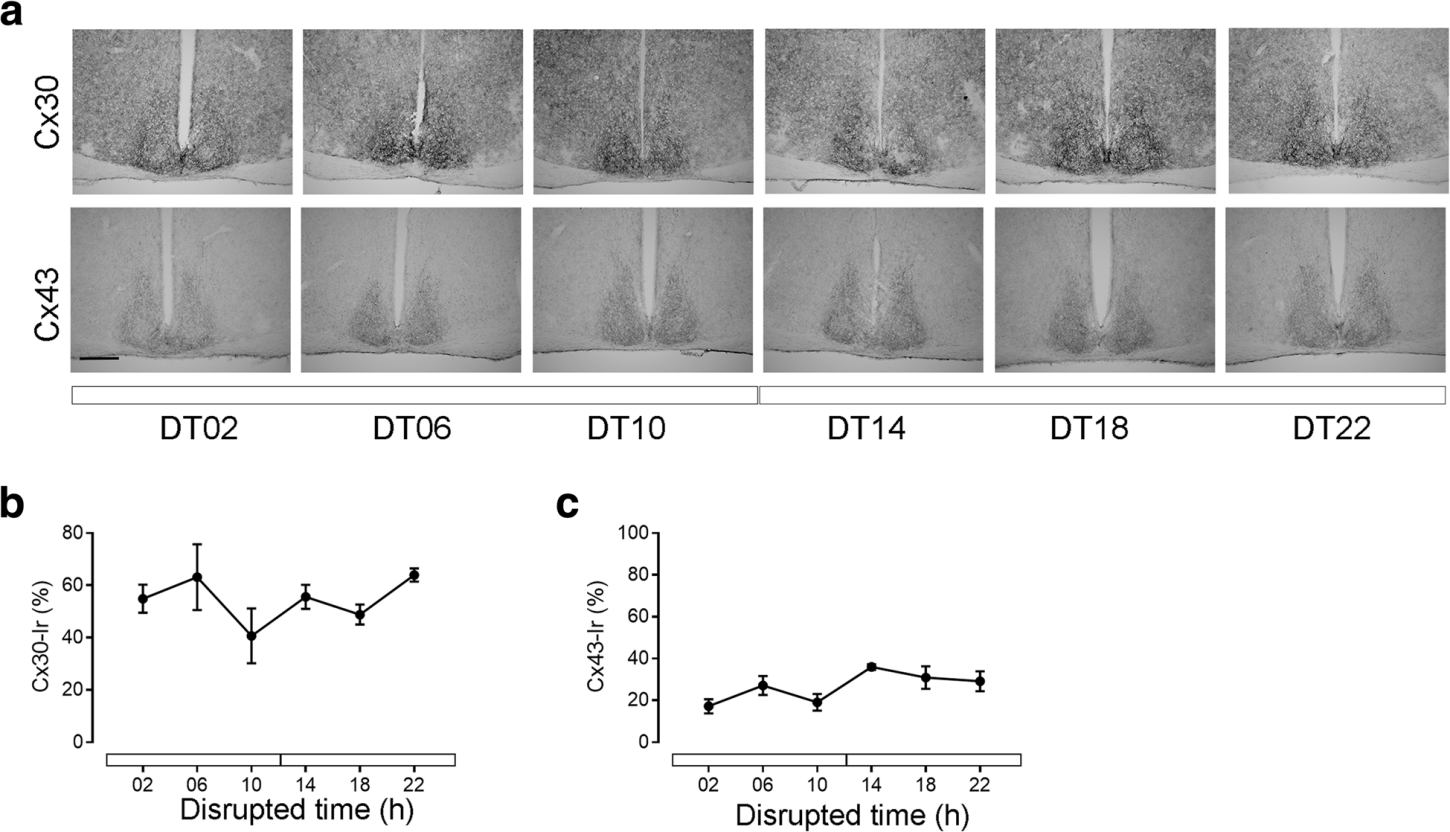
Cx43- and Cx30- immunoreaction in the SCN in constant light (LL). **a** Representative photomicrographs of Cx30- and Cx43-Immunoreaction (Ir) in the SCN of C57Bl/6 mice kept under LL. Scale bar: 300 μm. **b** Quantification of Cx30-Ir in the SCN. **c** Quantification of Cx43-Ir in the SCN. Data are expressed as mean +/− SEM of n = 3 mice per time point. Significant differences among different time points were analysed by one way ANOVA. There were no significant differences in Cx30- or Cx43-Ir levels among different time points under LL conditions

Constant light (LL) is known to disturb circadian rhythms [34]. Therefore, animals were kept for 14d under constant light before measurements. In LL, Cx30-Ir in the SCN showed a high level at all time points (Fig. 4a, b) whereas Cx43-Ir showed a low level at all time points (Fig. 4a, c).

### Locomotor activity in Cx30/Cx43 double deficient mice

**Fig. 5:**
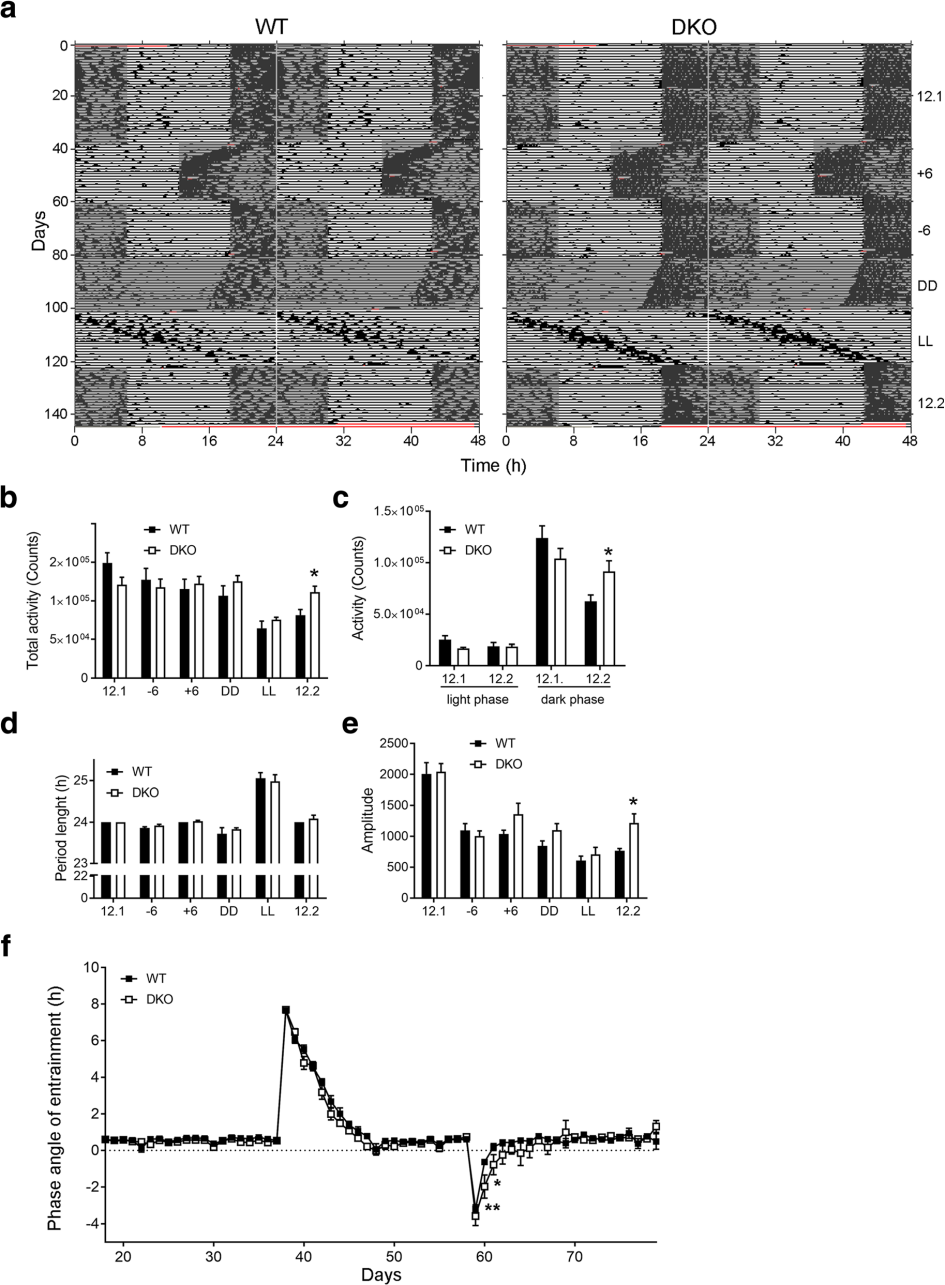
Locomotor activity in Cx30/Cx43 double deficient mice. **a** Representative double-plotted actograms of spontaneous locomotor activity of a WT and a DKO mouse under different light regimes: 12:12 LD (12.1), 6 h advanced phase shift (+ 6), 6 h delayed phase shift (− 6), constant darkness (DD), constant light (LL) and second 12:12 LD (12.2). Black bars show spontaneous locomotor activity in 10 min bins. Grey boxes indicate dark phases. **b** Analysis of total activity under different light regimes. In LL, total activity was reduced in both, WT and DKO. Activity levels were not different between both genotypes when mice were kept in 12.1, + 6, − 6, DD, LL. However, in 12.2, the total activity was significantly higher in DKO as compared to WT (F = 1.28; T-Test: t = 2.48, *P* = 0.017). **c** Analysis of activity counts of WT and DKO during the light and dark phase in 12.1 and 12.2. In 12.2, activity during the dark phase was lower in WT as compared to DKO (F = 3.23; t-Test: t = 2.26, *P* = 0.04).**d** Analysis of the circadian period length under different light regimes showed no significant difference between the genotypes. **e** Analysis of amplitudes of circadian rhythms under different light regimes. There was no significant difference in amplitude between the genotypes in any of the light regimes. In 12.2 the amplitude was significantly higher in DKO as compared to WT (F = 20.09, Mann Whitney test, *P* = 0.018). Data are expressed as mean + SEM. **f** Phase angle of entrainment in 12.1, phase advance (+ 6) and phase delay (− 6) is defined as activity onset relative to light off. Phase angle of entrainment in 12.1 and after phase advance (+ 6) was not different between both genotypes. However, in first (F = 38.43, Mann Whitney test, *P* = 0.007) and second day (F = 11.12, Mann Whitney test; *P* = 0.046) after phase delay (− 6), the phase angle of entrainment was significantly higher in DKO as compared to WT Data are expressed as mean +/− SEM of *n* = 6 WT and *n* = 7 DKO mice. *P < 0.05, **P < 0.01 WT vs. DKO mice

Spontaneous locomotor activity was measured in WT mice and DKO mice under different light regimes consisting of: a first cycle of 12:12 LD (12.1, 3 weeks), 6 h advanced phase shift (− 6, 3 weeks), 6 h delayed phase shift (+ 6, 3 weeks), constant darkness (DD, 3 weeks), constant light (LL, 3 weeks), and 12:12 LD (12.2, 3 weeks) (Fig. 5a). Under the first cycle of LD (12.1), both WT and DKO showed a significantly higher spontaneous locomotor activity during the dark phase as compared to the light phase This shows entrainment of locomotor activity to the dark phase is not affected by connexin-deficiency. In both, WT (paired T-test, t = 6.87, *P* = 0.001) and DKO (paired T-test, t = 4.49, *P* = 0.0007) mice, total activity was higher in 12.1 as compared to LL (Fig. 5b). This shows a damping of activity during chronodisruption. However, only in WT (paired T-test; t = 9.25, *P* = 0.0002) but not in DKO mice, total activity was higher in 12.1 as compared to 12.2 (Fig. 5b). This shows that damping of activity after chronodisruption persists in WT mice and that DKO mice can compensate for damped nocturnal activity after chronodisruption. In 12.1 and 12.2, both WT (paired T-test; 12.1 light/dark: t = 8.51, *P* = 0.0004; 12.2 light/dark: t = 6.16, *P* = 0.0016) and DKO (paired T-test; 12.1 light/dark: t = 8.51, *P* = 0.0001; 12.2 light/dark: t = 5.82, *P* = 0.0011) mice show a higher activity during the dark phase as compared to the light phase (Fig. 5c). This shows that locomotor activity is entrained to the dark phase. This was consistent with a significantly higher cFOS-Ir at ZT02 as compared to ZT14 in the SCN both genotypes (Additional file 2: Figure S2) in the SCN of both genotypes. In 12.2 WT mice showed a lower activity during the dark phase as compared to DKO mice (5C). Period length (Fig. 5d) was not significantly different between WT and DKO mice at any light regime indicating that circadian period is not affected by the connexin-deficiency. There was also no significant difference in amplitude between the genotypes in any of the light regimes except in 12.2, where the amplitude was significantly higher in DKO as compared to WT mice (F = 20.09, Mann Whitney test, *P* = 0.018), indicating a more stable rhythm in DKO mice (Fig. 5e). However, one and two days after the 6 h phase delay, the phase angle of entrainment was significantly larger in DKO mice as compared to WT mice (Fig. 5f). This shows that re-entrainment after phase delay is slightly affected by connexin-deficiency.

## Discussion

Astrocytes have been shown to express clock genes, and exhibit circadian oscillations even without neuronal interaction [35]. Moreover, expression of the astrocytic markers GFAP [36–38] and Ezrin [39] in the SCN shows time-of-day dependent changes. In the SCN, astrocytes play a pivotal role in both, photic entrainment (Leone, et al. 2015) and circadian timekeeping (Brancaccio, et al. 2017) by controlling extracellular glutamate levels. Recently, it has been shown that the astrocytic clock drives rhythmic neuronal function in the SCN via rhythmic glutamatergic signals and this mechanism can reinstate and maintain rhythmic behaviour in arrhythmic mice [40]. Glial Cx30 and Cx43, as well as neuronal Cx36, are expressed in the SCN [27]. However, it is not known so far whether expression of Cx30 and Cx43 in the SCN shows time of day-dependent changes. Also, the role of these astrocytic gap junction proteins in entrainment and generation of circadian rhythms is poorly understood. There is some evidence that Cx43 is involved in rhythmic astrocytic clock gene expression and in the rhythmic release of glutamate from astrocytes [40]. In this study, we have analysed the time-of-day dependent expression of Cx30 and Cx43 in the SCN. In addition, we used DKO mice to see how a complete disruption of astrocytic coupling affects the rhythm in spontaneous locomotor activity under different light regimes.

Cx30 and Cx43 transcript as well as protein levels exhibit a light-dependent variation in the SCN. However, DKO mice show a regular entrainment and a regular circadian rhythm of spontaneous locomotor activity. Only in an experimental jet lag paradigm, DKO mice do not re-entrain as fast as the WT mice to a 6 h phase delay. This suggests that glial Cx43 and global Cx30 are dispensable for circadian entrainment and rhythm generation in mice with intact neuronal molecular clockwork. However, they facilitate entrainment and maintain rhythm stability under challenging conditions such as jetlag or constant light, probably through modulation of synaptic activity and plasticity [13] as well as rhythmic glutamate release [40].

In the SCN of mice kept in LD12:12, *Cx30* and *Cx43* mRNA expression shows a time-of-day dependent fluctuation with peak levels during the early dark phase (ZT14) and low levels during the late dark phase (ZT22). In contrast, there was no fluctuation of *Cx30* or *Cx43* mRNA expression in the SCN of mice kept in constant darkness. Clock genes such as *per1* and *tim* [41, 42] or clock controlled genes such as *dbp* [43] show peak expression levels during the light phase and their rhythms persist in constant darkness. Thus, it seems unlikely, that *Cx30* and astroglial *Cx43* mRNA expression in SCN is tightly controlled by the molecular clockwork. However, in the urinary bladder, transcription of *Cx43* is regulated by the circadian clock component Rev-erbα [44]. Similarly, rhythmic melatonin synthesis persists in constant darkness [45]. Moreover, C57BL/6 mice kept in constant darkness do not show a significant increase in melatonin during the dark phase [46]. Thus it is also unlikely, that that *Cx30* and *Cx43* mRNA expression is controlled by melatonin.

In LD, both Cx30-Ir and Cx43-Ir show a time-of-day dependent variation with higher levels during the light phase and the early dark phase and low levels during the mid-to-late dark phase. Cx43 is subject to post-translational modifications, such as phosphorylation, modulating its turnover and function [47]. Phosphorylation-inducing kinases such as MAPK, PKA [48] and PKG [49], play an important role in the light-entrainment of the SCN circadian clock. In addition, Cx43 degradation is tightly controlled by post-translational modifications including MAPK pathway induced phosphorylation [47]. Interestingly, MAPK activity displays a circadian rhythm [50]; therefore, it is possible that it drives, subsequently, a circadian alteration in post-translational modification of Cx43. Thus, the discrepancy in the temporal profile between Cx mRNA and protein expression might be due to post-translational modification that modulates the life cycle of Cx43. As Cx43 is involved in astrocyte-neuron metabolic interactions [22, 40], the fluctuation of Cx43 may have modulatory contribution to SCN neuronal function. However, for electric coupling between SCN neurons Cx36 is necessary [28]. Here, we show that in the rodent SCN not only Cx32 and Cx43 [51] but also Cx30 and Cx43 are expressed. Both, Cx30 and Cx43 can form hemichannels, which might be involved in the release of ATP [52]. The rodent SCN shows a circadian rhythm in accumulation of ATP in the extracellular space [53]. However, the peak in ATP accumulation during the middle of the dark phase [53] is in antiphase to the peak in Cx30- and Cx43-Ir observed in this study. Thus, it seems unlikely that rhythmic Cx30 or Cx43 expression drives rhythmic ATP accumulation in the SCN.

In DD, the overall levels of Cx30-Ir were reduced as compared to LD and not significantly different among the different time points. This suggests that the oscillation of Cx30 protein levels strongly depends on the light/dark cycle. This is reminiscent of glutamate uptake in the SCN which is faster during the day as compared during the night but not different under DD conditions [54]. It is tempting to speculate that glutamate release from the retinohypothalamic tract during the day/light phase stimulates synthesis, transport and/or integration of Cx30 into the membrane. Exposure to constant light disrupts the circadian organization and alters proteins expression in SCN leading to arrythmicity [55]. Consistently, Cx30-Ir was not different among time points in LL. Moreover, the overall levels of Cx30-Ir were increased as compared to LD, supporting the hypothesis of a stimulatory effect of light on Cx30 expression. In contrast, the overall levels of Cx43-Ir were reduced in LL. This suggests that Cx30 and Cx43 are regulated differently.

Cx43-Ir was significantly higher during the early subjective day as compared to the late subjective night in DD. This suggests that Cx43 protein expression is under the control of the circadian clock to some extent. This rhythm might be due to post-translational modification of Cx43 as mRNA levels did not show a circadian rhythm. SCN electrical activity [56] and glutamine synthase activity [54] are high during the day/subjective day, independent of light input. An association between expression of Cx43 and glutamine synthase activity has been described [57]. Thus, synthesis, transport and/or integration of Cx43 into the membrane could be coupled to SCN neuronal activity and/or glutamine synthase activity.

Interestingly, a variety of tissues show circadian rhythms in connexins expression such as retina (Cx36) [58] and heart (Cx40, Cx43) (Tong et al., 2016). The activity of some connexins seems to be also time-of-day-dependent, as the phosphorylation state of Cx36 exhibits circadian oscillation [59].

To test the role of Cx30 and glial Cx43 in the generation and synchronization of circadian rhythms, we analysed spontaneous locomotor activity of DKO mice under different light conditions. Under the first LD cycle (12.1), level of spontaneous locomotor activity in both DKO and WT was comparable; indicating that regulation of motor activity is not affected in DKO mice. Moreover, DKO mice entrain perfectly to the regular LD cycle. Consistently cFOS-Ir in the SCN, which is known to be induced by light/glutamate [60], is high during the early light phase and low during the early dark phase in both WT and DKO mice. Thus, Cx30 and glial Cx43 seem to be dispensable under regular LD cycle for the entrainment of circadian rhythms. A recent study reported a decreased activity Cx43 cKO^GFAP^ mice [22] during the active phase. In contrast, DKO mice did not show a decreased activity during the active phase under a regular LD (12.1) cycle. Thus, reduction of activity as a consequence of glial Cx43-deficiency might be compensated by Cx30-deficiency. Moreover, in LD (12.2), after LL-induced chonodisruption [61], DKO mice showed a significantly higher activity during the active phase as compared to WT associated with higher amplitude of the rhythm. This suggests that DKO mice are more resilient to chronodisruption. This is consistent with the effect of a central-acting connexin inhibitor in preventing a feeding pattern disturbance induced by high-fat diet [62].

Period length and amplitude of circadian rhythm were not different between WT and DKO mice kept in DD. This shows that glial Cx43 and Cx30 are dispensable for circadian rhythm generation. This is in contrast to changes in period length of circadian rhythms in locomotor activity observed in Cx36-deficient mice [28, 29]. Thus, in the SNC Cx36-mediated neuronal coupling is functionally more important than glial coupling through Cx30 or Cx43. Interestingly, re-entrainment after a 6 h phase delay (− 6) following a 6 h phase advance (+ 6) in a jet lag experiment, was faster in WT as in DKO mice. This suggests, that re-entrainment is slightly impaired by Cx43/Cx30-deficiency under challenging conditions.

## Conclusion

In summary, *Cx30* and *Cx43* mRNA show a time-of-day dependent expression in the SCN with peaks during the early dark phase. The peaks are absent in constant darkness. Cx30- and Cx43-Ir show a time-of-day dependent variation in the SCN with high levels during the light phase. In constant darkness, only Cx43-Ir shows a significant variation. In constant light, Cx30-Ir is constitutively high, whereas Cx43-Ir is constitutively low. These findings suggest that Cx30 and Cx43 are differentially regulated. Mice with disrupted astrocytic coupling (DKO) entrain to a regular light/dark cycle and show regular amplitude and period length of circadian rhythm in spontaneous locomotor activity. However, nocturnal activity in DKO mice is more resilient to previous chronodisruption and re-entrainment in a jet lag paradigm is slightly affected. Thus, our findings provide a novel role of astrocytic coupling in modulating nocturnal activity and re-entrainment under challenging conditions.

## Additional files

Additional file 1: **Figure S1.** Validation of Cx30 and Cx43 antibodies. Representative photomicrograph of Cx30-immunoreaction (Ir) in (A) coronal brain sections and (B) the SCN of WT and DKO mice. Representative photomicrograph of Cx43-Ir in (C) coronal brain sections and (C) the SCN of WT and DKO mice. OC = optic chiasm. (A, C): Scale bar: 500 μm; (B, D): Scale bar: 100 μm. (TIF 107072 kb)

Additional file 2: **Figure S2.** Day/night variation of cFOS-immunoreaction (Ir) in SCN. (A) Representative light photomicrograph of cFOS-Ir at the early light phase (ZT02) and the early dark phase (ZT14) in SCN of WT and DKO mice. Scale bar: 300 μm. (B) Analysis of cFOS-Ir in the SCN. In both WT (F = 1.1; *P* = 0.02) and DKO (F = 1.7, *P* = 0.04) the level of cFOS-Ir is higher at ZT02 as compared to ZT14. There is no difference in cFOS-Ir between WT and DKO at ZT02 (F = 1.3, *P* = 0.08) or at ZT14 (F = 1.15, *P* = 0.43). Data are expressed as mean + SEM of *n* = 5 WT and *n* = 6 DKO mice per time point. (TIF 8506 kb)

## Abbreviations

12.1: First cycle under 12 h light/ 12 h darkness
12.2: Second cycle under 12 h light/ 12 h darkness
CT: Circadian time
Cx30: Connexin30
Cx43: Connexin43
DD: Constant darkness
DKO: Cx43fl/fl: hGFAP-Cre/Cx30−/− mice
DT: Disrupted Time
Ir: immunoreaction
LL: Constant light
PBS: Phosphate buffered saline
qPCR: Quantitative polymerase chain reaction
Rn18S: 18S ribosomal ribonucleic acid
SCN: Suprachiasmatic nucleus
WT: Wildtype mice
ZT: Zeitgeber Time
